# Complications arising from a misdiagnosed giant lipoma of the hand and palm: a case report

**DOI:** 10.1186/1752-1947-5-552

**Published:** 2011-11-15

**Authors:** Thomas Pagonis, Panagiotis Givissis, Anastasios Christodoulou

**Affiliations:** 1 1st Orthopedic Department of Aristotle's University of Thessaloniki, General University Hospital 'Georgios Papanikolaou', Thessaloniki, Greece

## Abstract

**Introduction:**

Lipomas are benign tumors which may appear in almost any human organ. Their diagnosis rate in the hand region is not known.

**Case Presentation:**

We present the case of a 63-year-old Greek Caucasian woman with a giant lipoma of the hand and palm which was not initially diagnosed. After repeated surgical decompression of the carpal tunnel the patient was referred with persisting symptoms of median and ulnar nerve compression and a prominent mass of her left palm and thenar eminence. Clinical examination, magnetic resonance imaging, nerve conduction study and biopsy, revealed a giant lipoma in the deep palmar space (8.0 × 4.0 × 3.75 cm), which was also infiltrating the carpal tunnel. She had already undergone two operations for carpal tunnel syndrome with no relief of her symptoms and she also ended up with a severed flexor pollicis longus tendon. Definitive treatment was performed by marginal resection of the lipoma and restoration of the flexor pollicis longus with an intercalated graft harvested from the palmaris longus. Thirty months after surgery the patient had a fully functional hand without any neurological deficit.

**Conclusion:**

Not all lipomas of the wrist and hand are diagnosed. Our report tries to emphasize the hidden danger of lipomas in cases with carpal tunnel symptoms. The need for a high index of suspicion in conjunction with good clinical evaluation and the use of appropriate investigative studies is mandatory in order to avoid unnecessary operations and complications. Marginal excision of these tumors is restorative.

## Introduction

Lipomas are the single most common soft tissue tumor [1]. However, their presentation in the hand is infrequent. Some lipomas can grow considerably and their presence in the hand is associated with a variety of symptoms. Giant lipomas exhibit a size of more than 5 cm. In this anatomical location they may cause a multitude of symptoms due to local compression of the surrounding tissues [2,3]. Grasping activities can be compromised due to the lipoma's considerable size. Lipomas, however, may present as liposarcomas, which are the most common soft tissue sarcomas. Their largest subgroup is the well-differentiated liposarcomas, which account for 40% of cases and require a different therapeutic approach. We report the case of a patient with a giant lipoma of the deep palmar space that was misdiagnosed and mistreated leading to severe complications. We emphasize the need for marginal surgical removal due to complications from the lipoma's occupation of palmar space that created subsequent nerve compression symptoms. These misdiagnosed symptoms originally led the first treating physicians to a false diagnosis of a regular carpal tunnel syndrome with two unsuccessful surgical decompressions further complicated by a severed flexor pollicis longus tendon.

## Case Presentation

A 63-year-old Greek Caucasian woman was referred with symptoms of median and ulnar nerve compression and a prominent mass on her left palm and thenar eminence to the outpatient clinic of our upper limb and hand surgery unit. She already had had two operations for carpal tunnel release within the last three years in another hospital and despite that, her situation had worsened.

**Figure 1:**
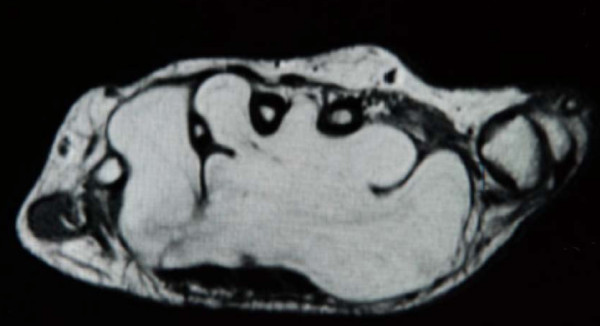
**MRI showing the recesses of the lipoma, invading the web spaces between the metacarpals**.

**Figure 2:**
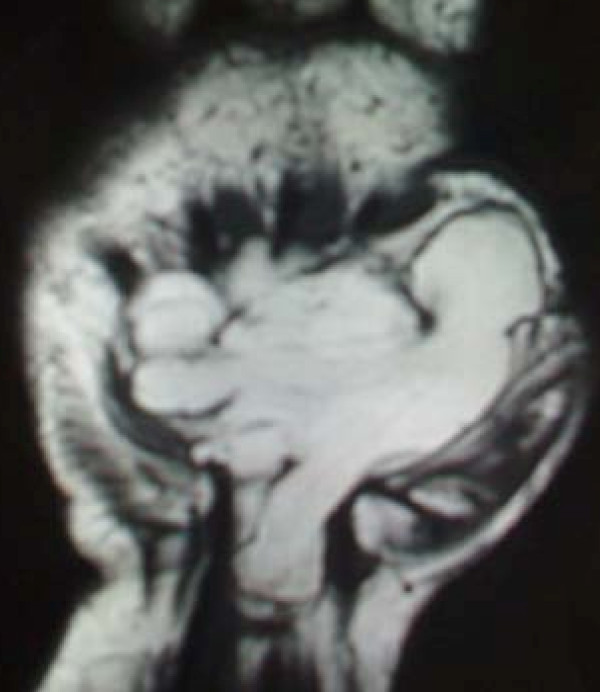
**MRI picture showing one of the recesses invading the carpal tunnel**.

Physical examination revealed a soft tissue mass which was slightly tender on palpation, with a diminished range of motion of the wrist (extension 40°, flexion 60°). She was also unable to flex the distal phalanx of the thumb. The mass was apparent on plain X-rays and an MRI examination showed a well-orientated tumor, measuring 8.0 × 4.0 × 3.75 cm. It extended from the deep palmar space, between the tendons and the metacarpals leaving the periosteum intact (Figures 1 and 2). The mass was hyper intense in T1 and had low signal in fat suppression, which was suggestive of an inter-intra muscular lipoma, the so-called infiltrating lipoma which was confirmed by biopsy. A nerve conduction study performed on the anatomically involved nerves showed alteration of the normal values due to pressure on both the median and ulnar nerves.

**Figure 3:**
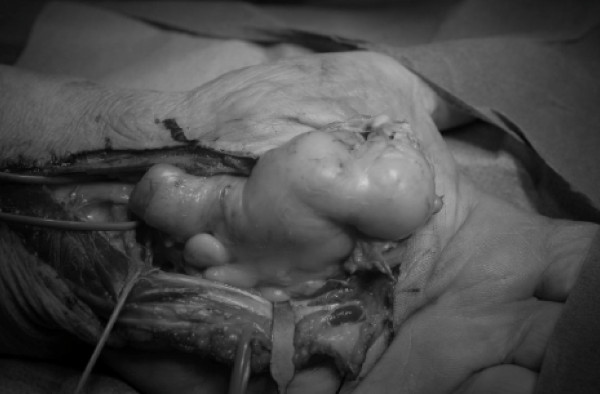
**The lipoma was excised en block**.

**Figure 4:**
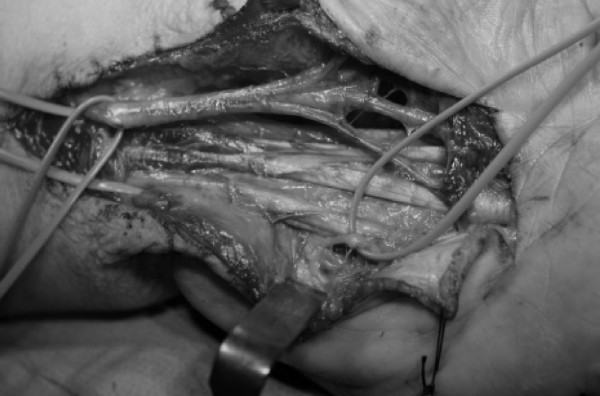
**The flexor tendons and the median and ulnar nerves are identified and protected**.

**Figure 5:**
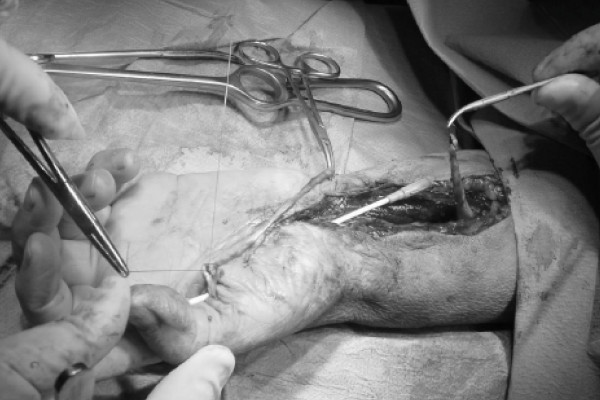
**Reconstruction of the Flexor Policis Longus tendon with an intercalated autograft of Palmaris Longus tendon**.

Surgery was performed under axillary block and by tourniquet controlled hemostasis. A palmar zig-zag incision was made. The tumor was identified and found to compress all the surrounding tissues. Intra-operatively, the median and ulnar nerves were carefully identified and protected. Marginal excision of the tumor was performed which decreased inter-compartmental pressure which had previously affected all relevant anatomic structures (Figures 3 and 4). Intra-operatively, we discovered that the flexor pollicis longus tendon had been severed in one of the previous carpal tunnel release operations. We decided to reconstruct the tendon in a second phase operation, four months later with an intercalated palmaris longus autograft (Figure 5). Histopathological analysis of the resected tumor revealed a mature adipose tissue consistent with a lipoma, and no evidence of any malignancy.

At the final follow-up thirty months postoperatively, the hand was pain-free without neurological deficit and exhibiting full range of motion. Thumb motion exhibited no deficits.

## Discussion

Lipomas consist of mature fat cells, which may occur in subcutaneous, inter-muscular or intra-muscular locations. They generally progress slowly and painfully which explains their often large size at diagnosis, particularly if located deeply. The differential diagnosis between lipomas and well-differentiated liposarcomas is extremely important and allows appropriate monitoring and treatment planning. Recently, many cytogenetic studies have dealt with tumors originating from adipocytes, including benign lipomas, as well as malignant well-differentiated liposarcomas (WDLPS). The majority of these tumors present genetic mutations in regions 12q13-15 and 6p13q [4]. Any soft tissue mass larger than 5 cm should be regarded as malignant until proved otherwise [5]. The term well-differentiated liposarcoma has been used to describe low-grade lipomatous neoplasms with a propensity for local recurrence [6]. Well-differentiated lipoma-like liposarcomas are one of the more common subtypes of the well differentiated liposarcomas. It is doubtful whether liposarcomas ever differentiate from pre-existing lipomas [2].

Adipose tissue is so widely distributed throughout the human body that one would expect these tumors to be one of the most frequently encountered benign neoplasms. The small numbers of cases being treated for this kind of tumor might be an indication of a high rate of misdiagnosis. Apart from cosmetic reasons the only indication for surgical treatment is the existence of complications arising from compression of local neurovascular structures which is a high occurrence in the narrow spaces within the fascial sheaths demarcating the regions of the hand.

Soft tissue lipomas are categorized by anatomic location as either superficial (subcutaneous) or deep and their contour is determined by the confines of the space the tumor occupies. When there is no limiting fascia, muscle, bone or organ, the lipoma is usually round. Fat tumors however have the ability to insinuate themselves into small recesses and thus produce tumors of any size or shape, infiltrating spaces not tightly guarded by protecting sheaths as fascia. This is especially true with tumors of the hand where lipomas occur in various anatomic locations within it. Superficial lipomas arise in the subcutaneous tissues while deep lipomas arise in the Guyon's canal, in the carpal tunnel, and the deep palmar space. Brand and Gelberman [7] advocated the addition of deep palmar lipomas to the list of the possible causes of carpal tunnel syndrome as was the case with our patient. The deep-embedded and intramuscular lipomas are less defined, considerably larger in size, and much less common than their superficial counterparts [8] due to the thick palmar fascia obscuring the true size and extent of these tumors. Consequently, the required surgery may be more extensive than originally planned, due to the anatomy usually being distorted. Good results can be obtained with surgical treatment, but, as with large tumors located elsewhere, these require a thorough pre-operative assessment [9]. MRI scan is very helpful in planning surgery as it clearly shows the extent of the tumor and its relation to important structures [10]. A previous analysis of 134 cases revealed that MRI gave the correct diagnosis in 94% of cases [1]. The tumors lie in close approximation to important neurovascular structures and tendons, which makes the operation very demanding.

## Conclusion

It must be realized that not all lipomas of the wrist and hand are diagnosed. Our report emphasizes the hidden dangers of misdiagnosing or altogether missing lipomas in cases with carpal tunnel symptoms.

The need for a thorough clinical examination and a sound intra-operative investigation of all anatomical structures involved in the carpal tunnel release operation is of paramount importance. Although treating physicians may consider this type of operation a simple one, a high suspicion index concerning tissue involvement in conjunction with good clinical evaluation and the use of appropriate investigative studies are mandatory in order to avoid unnecessary re-operations and complications. This case presentation shows not only that a sound original diagnosis might have prevented three further re-operations and a complication including the flexor pollicis longus tendon, but also points out the necessity of a structured and complete clinical examination.

Our report stresses the fact that marginal excision of lipomas is restorative.

## Consent

Written informed consent was obtained from the patient for publication of this case report and any accompanying images. A copy of the written consent is available for review by the Editor-in-Chief of this journal.

## Competing interests

The authors declare that they have no competing interests.

## Authors' contributions

TP assisted in the surgery, analyzed and interpreted the patient data and was a major contributor in writing the manuscript. PG performed the surgery and was a major contributor in writing the manuscript. AC was a major contributor in writing the manuscript and performed the histologic analysis of the excised tumor. All authors read and approved the final manuscript.
